# Digital mental health treatment implementation playbook: successful practices from implementation experiences in American healthcare organizations

**DOI:** 10.3389/fdgth.2025.1509387

**Published:** 2025-02-11

**Authors:** David C. Mohr, Alexandra L. Silverman, Soo Jeong Youn, Patricia Areán, Andrew Bertagnolli, Jenna Carl, Tarolyn Carlton, Neha Chaudhary, David Cooper, Shelly DeVito, Stephanie Eaneff, Megan Flom, Valerie L. Forman-Hoffman, Leanna Fortunato, Karen Franchino, Andrea K. Graham, Heidi Greenberger, Jessica Hauflaire, Benjamin Kaveladze, Rachel Kornfield, Kaylee P. Kruzan, Eric Kuhn, Carolyn MacIver, Frederick Muench, Regina Misch, Adrian Ortega, Lisa Palko, Derek Richards, Louisa Salhi, Jonathan Schremp, Eva Szigethy, Nathan Tatro, Bethany A. Teachman, Trina Histon

**Affiliations:** ^1^ Department of Preventive Medicine, Center for Behavioral Intervention Technologies, Northwestern University, Chicago, IL, United States; ^2^ Society for Digital Mental Health, Irvine, CA, United States; ^3^ Department of Psychiatry, Harvard Medical School, Boston, MA, United States; ^4^ Reliant Medical Group, OptumCare, Worcester, MA, United States; ^5^ Division of Services and Intervention Research, National Institute of Mental Health, Bethesda, MD, United States; ^6^ One Medical, Alliant University-San Francisco Bay, California School of Professional Psychology, San Francisco, CA, United States; ^7^ Big Health, San Francisco, CA, United States; ^8^ Development & Commercialization, Otsuka Pharmaceutical Inc, Princeton, NJ, United States; ^9^ Modern Health, San Francsico, CA, United States; ^10^ TelaDoc, Purchase, NY, United States; ^11^ Woebot Health, San Francisco, CA, United States; ^12^ American Psychological Association, Washington, DC, United States; ^13^ Care Management Institute, Mental Health and Wellness, Kaiser Permanente, Oakland, CA, United States; ^14^ AbleTo Inc., New York, NY, United States; ^15^ Digital Therapeutics Alliance, Arlington, VA, United States; ^16^ National Center for PTSD, U.S. Department of Veterans Affairs, Palo Alto, CA, United States; ^17^ Department of Psychiatry and Behavioral Sciences, Stanford University School of Medicine, Stanford, CA, United States; ^18^ Adaptive Health, Chicago, IL, United States; ^19^ Feinstein Institutes for Medical Research, Manhasset, NY, United States; ^20^ Kooth Digital Health Services, Chicago, IL, United States; ^21^ SilverCloud by Amwell, Dublin, Ireland; ^22^ School of Psychology, Trinity College Dublin, Dublin, Ireland; ^23^ Digital Health and Engagement, Banner Health, Phoenix, AZ, United States; ^24^ Pediatric Psychology and Psychiatry, Akron Children’s Hospital, Akron, OH, United States; ^25^ Department of Psychiatry and Medicine, University of Pittsburgh, Pittsburgh, PA, United States; ^26^ Mental Health America, Alexandria, VA, United States; ^27^ Department of Psychology, University of Virginia, Charlottesville, VA, United States

**Keywords:** digital mental health, digital therapeutics, implementation science, clinical workflow integration, key performance indicators

## Abstract

**Introduction:**

Digital mental health treatments (DMHTs) have begun to be implemented in some healthcare systems across the United States. These implementations are conducted as business arrangements. Thus, information on successful or unsuccessful implementations is not published or disseminated. This slows progress, as experiences and learnings are siloed within each organization, hindering or preventing learning across implementations and slowing the progress. To address this, the Society for Digital Mental Health established a DMHT Implementation Workgroup, with the goal of developing a DMHT Playbook that describes current best practices in DMHT implementation in American healthcare settings.

**Methods:**

The workgroup was comprised of representatives from 7 healthcare systems and 10 DMHT companies that have conducted implementations, along with other stakeholders and technical experts. The workgroup met virtually to discuss implementation of effective DMHT implementation processes and inform the development of an interview guide, which was then administered to another 20 key opinion leaders with DMHT implementation experience. Concepts and thematic constructs were extracted by experts in qualitative data analysis. These findings were discussed and refined by the Workgroup based on the Workgroup's experience.

**Results:**

The resulting playbook includes detailed methods, processes and procedures, representing practices that have been successful for implementing DMHTs in healthcare settings.

**Discussion:**

The workgroup recognizes that DMHT implementation is a rapidly evolving field. The successful practices for DMHT implementation described in this playbook may be useful for improving the efficiency of future DMHT implementations in American healthcare systems. However, the authors caution that as the field rapidly evolves, successful implementation practices will likely evolve as well.

## 1 Introduction

The prevalence of mental health conditions has risen without enough mental health providers to meet the increased need (1, 2). Less than half of U.S. adults with a mental health condition received any mental health services in 2023, and the unmet need for services is even greater among youth (3). The COVID-19 pandemic further strained an already fragile mental healthcare system in the US, and pushed healthcare system leadership, reimbursement policies, and government regulations to support a rapid shift to telehealth to meet patients’ needs for services (4, 5). However, numerous barriers persist in adequately addressing the mental health needs, including cost, inadequate insurance coverage, and lack of available services (3). Indeed, more than 160 million Americans live in Federally Designated Mental Health Provider Shortage Areas (6).

Interest in digital mental health treatments (DMHTs; e.g., mobile applications, virtual reality, chatbots) among healthcare systems and consumers was amplified curing the COVID pandemic with the integration of telemedicine, and has continued to increase in the post-pandemic era (7). There is a large evidence base showing the effectiveness of digital mental health treatments (DMHTs; e.g., mobile applications, virtual reality, chatbots), for a wide variety of common mental health conditions such as depression, generalized anxiety, social anxiety, and obsessive compulsive disorder (8), with some studies showing outcomes to be similar to psychotherapy (8, 9). The effects are stronger when human support is provided, and there is no difference in outcomes between clinician and non-clinician supporters (9, 10). Evidence for the effectiveness of DMHTs for severe mental health conditions such as psychosis or mania, while promising, is mixed (11–13).

Growing interest among healthcare systems has also been driven by DMHTs’ potential to circumvent many patient barriers to care (e.g., lack of time or transportation, reluctance to speak with a professional), and ability to be delivered at scale and at lower costs to individuals who might not otherwise have access to services (e.g., individuals in rural areas) (7, 14). DMHTs are beginning to be implemented in many countries around the world where reimbursement and regulatory systems have been established, including many European and Asia Pacific countries (15–19). Methods of implementation vary widely given great differences across healthcare systems and remains challenging. A key challenge in the American healthcare system has been the absence of reimbursement mechanisms and lack of clarity in regulatory practices (20). This may be beginning to change. The Center for Medicare and Medicaid Services (CMS) announced reimbursement codes beginning in 2025 for DMHT devices and support for products that have obtained clearance from the Food and Drug Administration (FDA). While it will likely take time for adoption by commercial payers, this represents a substantial step towards removing reimbursement barriers.

Despite these challenges, healthcare systems in the United States (particularly those that operate outside the fee-for-service model) have begun to implement DMHTs within their organizations. For example, Kaiser Permanente, which uses a payvider model (i.e., combining the role of healthcare payer and provider), implemented a mental health and wellness digital ecosystem of existing mobile apps as part of standard care (21). The Reliant Medical Group, a primary care group medical practice that was acquired by Optum Care in 2018, implemented a digital ecosystem of pre-vetted, evidence-based DMHTs that are now offered as a first line of treatment to patients with mild-to-moderate anxiety and depression (22).^1^ These and many other DMHT implementations are taking place as pilots and business arrangements between DMHT companies and healthcare systems, and often do not have dissemination or publication of results as a goal. With no dissemination, there is no opportunity to learn from the past successes and failures of others, which slows the integration of DMHTs into the American healthcare system.

The aim of this project, conducted under the Society for Digital Mental Health (SDMH), was to surface the experiences of American healthcare organizations and DMHT companies that had engaged in DMHT implementation efforts. These findings constitute an initial DMHT Implementation Playbook that provides preliminary guidance and options for DMHT implementation, based on the accumulated knowledge and experiences to date.

## 2 Methods

SDMH (https://societydmh.org) is a non-profit organization that brings together members across academic research, healthcare systems, payers, policy, DMH companies, and people with lived experience to address the challenges impacting broad implementation of DMHT in the United States. The organization's mission includes disseminating knowledge and research, supporting the equitable implementation of digital mental health services through collaboration with stakeholders, and promoting effective and sustainable policy. The SDMH organized an implementation workgroup, led by two co-chairs (T.H., D.C.M.), that included members from 7 healthcare organizations and 10 major DMHT companies that had implemented DMHTs, as well as representatives from other organizations or interested parties, including the American Psychological Association, Digital Therapeutics Alliance, and the National Institute of Mental Health. The workgroup initially met for three remote planning meetings (11/6/23, 12/4/23, 2/2/24) to review their experiences with DMHT implementation.

In the second phase of the workgroup, semi-structured interview guides were developed based on the workgroup's experience. Two separate interview guides [one for key opinion leaders (KOLs) representing healthcare systems and one for KOLs representing DHMT companies] were developed by the workgroup co-chairs, initially based on the information gathered in the remote planning sessions, and subsequently refined following feedback from two researchers with expertise in human-centered design and qualitative methods (R.K., K.K). The resulting interview guide was reviewed by workgroup members. The final healthcare system interview guide questions probed on KOLs’ experiences implementing DMHTs in healthcare systems, patient engagement, data storage and exchange, equity considerations, and business decisions. The DMHT company interview guide questions covered DMHT design, company experience with DMHT implementation, data exchange, and ethical considerations. Final versions of both interview guides are included in the Supplementary Material. Interviews were conducted with 20 KOLs representing healthcare systems (*n* = 9) and DMHT companies (*n* = 11) The 20 KOL interviews were conducted over Zoom from February to April 2024 by two members of the research team (B.K., A.O). Zoom was used to audio record each interview, and recordings were then transcribed by a professional transcriptionist.

We used conventional content analysis (23) to identify and characterize thematic categories across transcripts. Each transcript was reviewed by 4 members of the research team (T.H., S.E., M.F., V.L.F-H.). For each of the concepts discussed in the interview guide, data were prepared in a structured format by copying relevant quotes into a spreadsheet. The complete codebook is included in the Supplementary Material of this publication. Once preliminary analysis was complete, the research team met to collaboratively review their coding results. Using a consensus-based approach, the team identified and defined a set of 3–10 thematic categories for each concept, including example quotes from participant(s) related to each theme. This work was determined to be non-human research by the Northwestern University Institutional Review Board.

Workgroup members then convened on May 3, 2024, for an in-person meeting to review and discuss results from the qualitative interviews. Results of the coding were provided to each workgroup member for review prior to the meeting. During the meeting, each concept and thematic category identified during the coding process was discussed. Workgroup members added any relevant information, refined the points, revised concepts to be presented in a playbook, and reached consensus on a maturity rating for each concept, reflecting the consistency of the findings and the level of confidence and agreement the group had in the reliability of the recommended processes (1 = low maturity, lack of agreement, processes ill-defined; 2 = moderate maturity, modest agreement, some clarity on processes; 3 = high maturity and agreement with clearly defined processes). Formal reporting methods such as Proctor's (24) were not used; actors, actions, and goals are described where information was or consensus was reached, however there was not sufficient information on others, such as outcomes, and implementers often did not describe a theoretical justification for their decisions or actions (24).

## 3 Results

All implementations to date have been for common mental health conditions such as depression, anxiety, or for subthreshold populations. There were no instances among workgroup members or KOL interviews in which DMHTs were used for severe mental illness.

### 3.1 Frameworks (maturity rating: 1, low)

Anchoring implementation in a theoretical framework allows partners to develop a clear understanding of intervention goals, identify target population/s, develop processes, and can support metric-based go/no-go decisions for expansion and scaling. Some workgroup members and KOL interviewees mentioned frameworks, including RE-AIM (25) and Quality Improvement frameworks (26), however others did not report using any formal framework. An evaluation guide created by the Digital Therapeutics Alliance (https://dtxalliance.org), a non-profit organization representing companies, was also mentioned as useful for clarifying the problem that the DMHT would be solving for, and the elements necessary to deploy the DMHT within healthcare settings (27).

There was discussion in the workgroup of blending these frameworks with Human Centered Design or design thinking. These use qualitative methods such as interviews, and shadowing provider and patient visits to identify problems, develop solutions, and optimize how new elements of care can be implemented. These methods are used both at the pre-implementation planning phase as well as during implementation to address problems (28).

### 3.2 Implementation stages (maturity: 3, high for pilot and scaling; 1, low for sustainment)

Prior to implementation, extensive exploration and planning must be conducted which includes all stakeholders, defines objectives and the measurement of objectives, and involves relevant stakeholders in the codesign of implementation processes. These pre-implementation processes are described throughout the results below. The workgroup identified three broad stages of DMHT implementation itself, with differing goals, evaluation metrics, and timeframes.

#### 3.2.1 Pilot implementation

Pilot refers to the initial small-scale testing of a DMHT within the healthcare system in a controlled environment. This stage involves introducing the DMHT to a limited group of patients and providers to gather data on the usability, effectiveness, and integration, and to refine implementation processes. For example, pilots can begin with a small number of providers or one site that is innovation-minded and monitor the implementation process closely for the first 25–50 referrals. Implementers track key metrics such as activations, usage rates, patient outcomes, and provider satisfaction. They meet regularly with providers and staff to understand what is working, what is not working, and solicit their input on potential fixes. This allows implementers to adjust and improve continuously using metrics and input from providers and staff. This process helps address the biggest problems before implementation is broadened to include additional providers and sites, and finally to scale the implementation.

#### 3.2.2 Scaling

Scaling refers to the process of expanding the DMHT beyond the initial pilot stage to reach a larger patient population within the healthcare system (29). The implementation strategies developed during the pilot are used, although they usually require continuing and iterative adaptation. During scaling, the DMHT is often integrated more deeply into standard care processes (for example, this is often when EHR integration occurs), and sustainable financial models are evaluated. Scaling up and out (30) is usually a staged process, expanding iteratively to a broader number of providers and settings, based upon a scaling plan that includes agreed upon check points when key milestones are reached (e.g., first 50 patients referred or the first 50 DMHT users activated). Scaling plans include timelines, training protocols and resource allocation ensuring each new location has access to training and expertise as they deploy. Careful planning is required to address challenges such as increased patient volume, provider workload, and technical support.

#### 3.2.3 Sustainability

Sustainability refers to the maintenance of the benefits and outcomes for patients, clinicians, and the healthcare system long after DMHTs have been fully implemented (31). Workgroup members recognized the importance of sustainment, however there were fewer examples, compared to pilots and scaling. Ensuring long-term sustainability by integrating the DMHT solution into regular clinical workflows and protocols requires ongoing reporting capabilities to ensure activations, engagement and outcomes are remaining consistent, over time. Ongoing provider and patient training and support materials also need refreshing as products iterate, mature, and add new features and functions. A sustainment plan should also recognize the changing landscape of healthcare systems, patient expectations and preferences, and DMHT products, and should include clear decision gates for removing DMHTs that are no longer delivering value to the healthcare organization.

### 3.3 Stakeholder engagement (maturity: 3—high)

Engagement of stakeholders is crucial to success throughout the pilot process. At the beginning, engagement of stakeholders is needed to obtain vertical alignment (alignment at all levels of the organization) on the value and goals of the pilot, and the development of the implementation plan. As the pilot progresses, engagement of stakeholders must be sustained to manage the implementation and the multiple challenges that will inevitably arise. Within the healthcare system, stakeholders include, but are not limited to, providers and staff from clinical services who must trust in the effectiveness and importance of the DMHT to the clinical mission; leadership and administration who must invest resources and promote adoption; compliance and legal who manage liability and risk; quality departments to ensure safety and effectiveness; information technology; and marketing to amplify across provider and patient channels. Many healthcare organizations also included patient representative groups in their stakeholder engagement to bring the voice of their patients or members to the table.

Once a DMHT company or companies have been selected, they should also be considered stakeholders in the pilot and integrated into processes where needed. In addition to understanding their products or services, companies often have experience from previous pilots that can inform the development of an implementation plan, which can provide unique experience in managing implementation processes.

Methods of engagement of stakeholders include (1) *Identification and mapping* of stakeholders, who would be impacted by or have a role in deploying or supporting the DMHT; (2) *Identification of leaders* of stakeholders who have influence and potential impact within their areas and can serve as champions; (3) *Establish consistent communication methods* through regular meetings and workgroups to facilitate the flow of information and decision-making; (4) *Establish input channels* for feedback from the broader interested party, tailored to their needs. This can include focus groups, surveys, or advisory groups.

We note that the importance of establishing and maintaining *trust* across all stakeholders was a consistent theme across discussion groups and KOL interviews. Trust can be difficult to achieve across all stakeholders and can be fragile over the course of implementation. The workgroup emphasized the importance of maintaining communication, transparency, respect, and involvement in decision making processes throughout the pilot. We mention issues related to trust throughout this document.

### 3.4 Implementation planning (maturity; 3-high)

An implementation plan is a document or set of documents that outlines the goals, processes, and procedures for the pilot and scaling (32). This planning document addresses the roles of people, processes and technologies, and is divided into phases or waves. The development of this document should be a collaborative venture, providing shared understanding of the goals, processes, and procedures. The document also serves to document the agreed-upon implementation plan. The plan can and should be updated to reflect changes as they arise.

We describe below the key decisions and processes required to develop the implementation plan and launch a pilot. There was consensus in the workgroup that it is not possible to do everything. Developing an implementation plan usually requires decisions about which goals and objectives to prioritize and which can be left for future work. Those decisions are informed by the broader vision a healthcare system may have for DMHT integration and what key information is needed for future decisions to scale, continue, or terminate.

### 3.5 Defining goals, key performance indicators (KPIs), and targets (maturity: 3)

*Goals* refer to the objective that the healthcare system is trying to achieve. *KPIs* are quantifiable indicators of progress towards an intended goal. *Targets* are the levels or benchmarks that you are aiming to achieve (33). Table 1 displays the primary goals and KPIs, along with examples.

**Table 1:** Digital mental health treatment (DMHT) implementation goals, Key performance indicators, and example metrics.

Goal	Key performance indicators	Example metrics
Improving mental health treatment access	Reach: Absolute number, proportion, and representativeness of people willing to engage with the DMHT	Number and proportion of DMHT account activations out of all patients referred
	DMHT usage: Assesses the rate of initial patient uptake of the DMHT, as well as various engagement metrics indicating the level of sustained engagement over the course of treatment	Proportion of patients referred to the DMHT who use it at least once; Number using ≥4 weeks; proportion activating or using ≥4 weeks relative of proportion of psychotherapy referrals initiating or in treatment ≥4 sessions.
	Reductions in wait times for mental health services	Compare time from referral to treatment initiation before and after DMHT implementation
	Health equity	Number and proportion of patients from underserved groups accessing the DMHT relative to proportions of underserved groups in healthcare system.
Treatment effectiveness and patient outcomes	Symptom severity: Changes in standardized patient-reported symptom severity over the course of treatment	Changes in PHQ-9 and GAD-7 scores from pre- to post-treatment; Change in PHQ-9 or GAD-7 for DMHTs relative to psychotherapy.
	Treatment response rates: Proportion of patients reaching a pre-established threshold indicating improvement in symptoms	For depression, proportion of patients using the DMHT with a PHQ-9 score ≤10 or ≤5 at post-treatment
	Patient safety & adverse events: Measures of any potential risks to the patient while using the DMHT, assessed via ongoing monitoring of suicidal thoughts and behaviors, hospitalizations, symptom deterioration, incident reports, patient complaints, and EHR audits	Psychiatric hospitalizations; fatal and non-fatal self-harm. Deterioration as measured by two consecutive PHQ-9 scores higher than pre-treatment score.
	Patient acceptability/satisfaction: The extent to which a DMHT or mental health service is perceived as satisfactory by patients	Number and proportion of patients agreeing to initiate DMHT. Patient ratings at post-treatment on a Client Satisfaction Questionnaire
Provider adoption and satisfaction	Provider Adoption: The number, proportion, and representativeness of providers and settings who refer to or deliver the DMHT	Number and proportion of providers making a referral to the DMHT out of all providers working in a setting
	Provider satisfaction and DMHT appropriateness: The extent to which a DMHT is perceived as acceptable, appropriate, feasible and level of satisfaction rated by providers and staff	Provider and staff ratings on a standardized measure such as the Acceptability of Intervention measure (34). Results of focus groups.
Financial performance	Cost savings	The cost of mental health services for those receiving the DMHT relative to a comparable group who received a standard treatment
	Revenue generated from the DMHT	Revenue generated through insurance reimbursement for the DMHT
	Return on investment	Ratio of the net return on investment relative to the cost of the DMHT services
	Reduction in avoidable or more costly healthcare services	Compare ER visits, inpatient hospitalizations, or frequent use of outpatient services from before vs. during the implementation
Other key performance indicators used to monitor implementation	Enhanced quality metrics	The proportion of patients screening positive for a mental health condition reaching recovery or remission
	Technical performance	Platform uptime and reliability; response times for technical support issues; data security and privacy compliance metrics
	Staff productivity	Number of patients seen by a providers or digital navigators over one week; Response times to patient questions; Time spent on administrative tasks
	Fidelity of human support services: The degree to which coaches are implementing human support services as intended	Number and length of coaching calls; Review of randomly selected coaching message logs or recorded calls

Goals, usually determined by leadership to support the healthcare system's overall objectives for healthcare quality and digital transformation, define the overarching purpose of the pilot and are used to identify KPIs. KPIs are used for two broad purposes. First, KPIs evaluate success, demonstrate value, and are used to inform decision-making as to whether the DMHT pilot should be scaled, sustained, or terminated. KPIs also support continuous quality improvement processes during the pilot, providing the data to monitor the success of the implementation strategies, identify challenges or problems, and develop strategies that address those inevitable challenges.

The process of defining KPIs should include a broad range of stakeholders from within the healthcare system, including but not necessarily limited to leadership, providers from relevant services, compliance, quality, information technology, innovation, business and finance, legal, and member representation. Companies often bring considerable experience and knowledge in implementing their products and can be useful in helping determine KPIs and targets. In addition, companies may have data collection interests, for example for improving their products, which should be considered in the interests of building a strong collaborative team. Common goals and KPIs included the following:

Improving access to mental health treatment is a common goal for DMHT implementations. KPIs include (1) *Reach*, which is defined as the absolute number, proportion, and representativeness of individuals willing to engage with the DMHT (35). (2) *DMHT use* is commonly measured to ensure uptake and sustained engagement and can be measured through metrics such as frequency of logins, time from first to last login, and completion rates for the program or modules. (3) *Reductions in wait times* for mental health services can be measured comparing time from referral to treatment initiation, using either historical data or data from similar services that are not part of the pilot as comparators. (4) *Health equity* KPIs can include the number and proportion of patients from underserved groups accessing the DMHT and geographic location of patients to evaluate access by patients in rural locations or locations that are far from clinics.

Treatment Effectiveness and Patient Outcomes evaluate how well the DMHT meets its therapeutic goals. KPIs can include (1) *Symptom severity* measured through standardized patient reported outcomes (e.g., the PHQ-9 or GAD-7) administered before, during, and after treatment by the healthcare system or collected through the DMHT. (2) *Treatment response rates* measured as the proportion of patients reaching a criterion (e.g., PHQ-9≤10 or ≤5 for depression); (3) *Patient safety and adverse* evaluates any potential risks to the patient and may be assessed through suicidal thoughts and behaviors, hospitalizations, deterioration (e.g., worsening of symptoms), incident reports, patient complaints, and EHR audits. (4) *Patient satisfaction* can be assessed through focus groups, interviews, and post-treatment questionnaires.

Provider Adoption and Satisfaction can use the following KPIs: (1) *Adoption* refers to the number, proportion, and representativeness of providers and settings who refer to or deliver the DMHT and can be evaluated by tracking the number of referrals or DHMT-related activities performed by each relevant provider or staff person. (2) *Provider and staff satisfaction* evaluate the acceptability, appropriateness, satisfaction, and feasibility of the implementation processes, and is commonly assessed through focus groups, questionnaires, and ongoing monitoring of processes and complaints (34, 36).

Financial performance was difficult to calculate in the United States given the few options for reimbursement at the time of this workgroup. KPIs included (1) *Cost savings,* calculated as the cost of mental health services for those receiving the DMHT relative to a comparable group who received a standard treatment. (2) *Return on investment* calculates the financial benefits overall to the healthcare system against the cost of the DMHT services. Participants noted, however, that the amount of time required to observe financial benefits or offset can be long and may exceed the length of the pilot. (3) *Revenue generated from the DMHT* is a potential KPI, however, the absence of uniform reimbursement mechanisms made this difficult. (4) *Reduction in avoidable or more costly healthcare services* can include any services, such as emergency room services or repeated use of outpatient services. Workgroup members noted that decreases in mental health services such as psychotherapy was a metric that was seen as potentially positive by healthcare systems in which a significant amount of expenditures are to contracted providers, but was viewed negatively for healthcare systems that use staff providers, as it could be seen as cannibalizing billable services.

Additional KPIs used to monitor the implementation include the following: (1) *Enhanced quality metrics* such as the proportion of patients screening positive for a mental health condition reaching recovery or remission. *Technical performance* indicators may include platform uptime and reliability, response times for technical support issues, data security and privacy compliance metrics, and data flow between the company and healthcare system. (2) *Staff productivity* indicators can monitor the efficiency with which staff (referrers, navigators, coaches, or others) are implementing the DMHT may include metrics such as the number of patients seen or contacts processed over a day or week, response times, or time spent in administrative tasks. (3) *Patients’ reasons for accepting or declining* the DMHT can help implementers understand implementation challenges and guide adjustments. (4) *Fidelity of human support services*, where applicable, can include the number and length of contacts as well as randomly selected ratings of messaging logs or recorded calls.

KPIs are generally collected, as much as possible, from data already captured as part of service delivery. Some KPI data may not be part of routine care, such as assessments of satisfaction, acceptability, appropriateness, and feasibility, or may not always be acquired with the frequency or reliability needed (for example, symptom severity measures integrated into DMHTs may not be consistently completed (33).

Targets are used to determine the completion of the pilot and scaling steps. This usually includes volume metrics, such as number of patients referred or registered with the DMHT. Some health healthcare systems also found it useful to set targets or *a priori* benchmarks that include the proportion of patients relative to the number of eligible patients, who are referred or activate the DMHT, the average length of time before treatment discontinuation, and the effectiveness of treatment (22).

In addition to being used to evaluate a pilot's success, targets can be helpful in prioritizing responses to the many challenges that may arise during implementation.

### 3.6 Product selection criteria (maturity level: 3-high)

Product selection criteria are used by the healthcare system to evaluate and choose a specific DMHT. There was consensus on the following evaluation factors: (1) *Alignment with health system goals* in meeting their ability to meet specific health system priorities and contributing to overall quality of care is typically the motivating factor in exploring DMHT options. For those that have already begun implementing digital health solutions, health systems also examine whether the product complements their existing portfolio of digital tools. (2) *Clinical efficacy* data from randomized controlled trials (RCTs) was universally required as it represents evidence that products can produce the desired clinical effects. RCT data is also required for FDA clearance, which is required for CMS reimbursement. However, it was generally recognized that research participants are often not representative of average patients. (3) *Clinical effectiveness or real-world data*, ideally collected from similar settings, is used to evaluate effectiveness and engagement among real-world patients. (4) *Company evaluation assesses* the maturity and stability of the company, and may include the funding stage, financial stability, and experience with implementation in similar settings. (5) *Data privacy and security* are an evolving and growing aspect of implementing DMHTs in healthcare, with focus areas including include being HIPAA compliance, clarity about user consent and transparency, encryption and data protection, access controls and authentication, data minimization to protect personal health information, and ongoing risk assessment and incident response and breach notifications per the Federal Trade Commission. These elements are typically addressed in contracting. Cybersecurity is a growing area of concern given the uptick in security concerns regarding data use, storage, and transmission, and healthcare is evolving their frameworks and expectations to acknowledge this growing area. (6) *Clinical review* of the DMHT product is often conducted by clinicians within the healthcare organization, who evaluate whether the product meets their criteria for acceptable inclusion of clinical content and use of psychological and behavior change principles. (7) *Health equity* has become increasingly important to healthcare systems. This evaluation can include reviews of inclusion of marginalized populations in RCTs, real world data with diverse populations, and review of product content for fit with the needs and preferences of diverse populations served by the healthcare system. In addition, healthcare systems commonly examine compliance with Americans with Disabilities Act requirements such as reading level or accessibility for people with disabilities. (8) *DMHT cost* is a consideration however, there are many pricing models. This is covered in the next section in more detail.

There were several product review criteria and processes that were mentioned by some, but not all healthcare systems. (1) *Product design processes* were mentioned by some, who were looking to see the use of human centered design processes that gave voice to end users in the design. (2) *Member or patient review* processes were used by some to evaluate the usability of the product. (3) *Interoperability,* the ability to integrate with existing information technology systems, was evaluated when such integration was considered part of the short-term or longer-term goals. (4) *FDA clearance* was a topic that generated considerable discussion and little agreement. At the time of the KOL interviews, it was not clear what role FDA clearance might play in any eventual reimbursement mechanisms, such as those recently announced by CMS.

### 3.7 Business models (maturity: 1-low)

Healthcare systems noted that they evaluated pricing models during the adoption phase to determine whether a proposed solution aligns with their budget, financial goals, and operational capacity. Although not addressed fully, the workgroup acknowledged that the length of the sales cycle is a known barrier to adoption and should be considered as companies enter this space. Acknowledging the time needed for relationship building and consensus within healthcare systems demonstrates an understanding of their decision-making dynamics.

#### 3.7.1 Pricing models

Pricing models are integral to the adoption process, as they help determine whether a digital mental health tool is financially viable and aligns with the healthcare system's goals. Value-based pricing, which ties costs to performance milestones like patient outcomes or cost savings, can create mutual incentives. However, these shared-risk contracts must avoid misalignments, such as holding companies accountable for metrics they do not control (e.g., Reach metrics when referral pathways are the responsibility of the healthcare system). Addressing these risks early in the decision-making process can foster trust and set the foundation for successful long-term collaboration.

#### 3.7.2 Sales cycle

The length of the sales cycle is an important factor for companies seeking to partner with healthcare systems, often lasting 12–24 months or longer. This reflects the complexity of healthcare decision-making, which involves multiple stakeholders, budget cycles, and organizational consensus-building. While challenging for companies seeking rapid growth, this extended timeline provides valuable opportunities to build trust, develop key relationships, and ensure alignment on shared goals. Recognizing the importance of this process as part of the adoption phase can lead to stronger long-term partnerships and smoother implementation.

### 3.8 Care pathways (maturity level: 2—moderate)

A care pathway is a structured multidisciplinary plan that outlines that defines what happens, when it happens, and who does what from diagnosis through treatment and follow-up for a defined population, designed to facilitate mutual decision-making and care provision to improve patient outcomes (37).

#### 3.8.1 Setting

The first step is to identify the clinical setting where the DMHT will be offered. There was broad agreement that the referral is best made in a care setting by a trusted provider. Direct-to-patient marketing through patient portal messaging, mailings, clinic flyers, or other general communications can build awareness of DMHT options and signal the healthcare organization's confidence in these as valid and effective treatment options. However, there was consensus among workgroup members and KOL interviewees that these direct-to-patient methods alone are unlikely to result in substantial uptake.

Many, but not all participants had found that DMHT uptake is best implemented in primary care (e.g., see text footnote 1) or medical specialty services. Several participants reported experiencing low patient uptake when implementing in mental health clinics, for example to patients on waitlists for psychotherapy. While offering to waitlists may be intended as a support, patients often feel they are being palmed off on a second-rate treatment. However, some reported good uptake through behavioral health services when initiation with a DMHT was presented by a behavioral care coordinator as the standard frontline treatment (22). Workgroup members speculated that the critical issue is to select a care setting where the patient has not yet made a decision about behavioral or psychological treatment; once the patient has decided to seek psychotherapy, they are less likely to be open to another option.

### 3.9 Clinical monitoring and evaluation

Clinical monitoring is based on measurement-based care, which is a process in which treatment outcomes are systematically monitored, feedback on progress is shared with the patient at regular time points, and the treatment plan is adjusted as needed until remission or recovery has been achieved. Measurement-based care is highly effective in improving population-level mental health outcomes and is recommended as a standard of care (38), yet it is estimated that fewer than 20% of providers use it (39). In the context of DMHTs, measurement-based care requires that assessment data are collected (which occurs in some but not all DMHT products) and that a provider reviews that data and can work with the patient to make treatment plan adjustments.

While there was agreement regarding the importance of measurement-based care, DMHT implementations varied their use of those processes. Some pilots had clearly identified staff, usually care managers or behavioral health specialists, who either collected patient reported outcomes directly from the patient for purposes of monitoring or reviewed data provided by the DMHT company. Other pilots used no formal processes for monitoring patient progress. While there was broad consensus that measurement-based care processes are valuable at improving outcomes, they can also require considerable investment in resources, including provider and staff time, training, and reorganization of workflows, which was beyond the scope of some pilots.

Pilots that monitored patient outcomes also monitored patient DMHT use. If a patient was not using the DMHT, the care manager or behavioral health specialist would contact the patient to problem solve to help the patient engage, help find another DMHT that was more suitable or connect the patient with another form of treatment.

#### 3.9.1 Post-treatment triage

Some portion of patients can be expected not to respond adequately to a DMHT. Those pilots in which care managers or behavioral health specialists monitored patient response, also included processes for identifying patients who were insufficiently responsive and initiated a process to refer them to another level of care. This process includes (1) a definition of adequate response (e.g., PHQ-9≤10); (2) a time window within which that response would occur; and (3) a process for referring the patient to another treatment (e.g., referring back to the referring provider or to mental health services). Similar to monitoring, this was beyond the scope of some pilots.

### 3.10 Referral pathways (maturity level: 2-moderate)

A referral pathway is a defined set of steps that will connect a patient with the DMHT service. While referral pathways can be considered part of a care pathway, making a successful referral is particularly challenging and is therefore presented separately. There was consensus, consistent with research, that referral by a trusted provider greatly increases the likelihood that the patient will initiate the DMHT (40). However, the referral juncture is both critical and fragile, with competing requirements. On the one hand, providers are already overburdened. On the other hand, many patients require attention to successfully engage with a DMHT. This is not unique to DMHT; only around 10% of primary care patients referred for psychotherapy ever follow up (41). Balancing provider burden and patient support is the central challenge at the referral juncture. We cover the referral steps here; the processes taken by providers and staff to promote engagement are covered under *Patient Engagement*.

#### 3.10.1 Trigger

The provider identifies that the patient has the mental health condition and is suitable for the DMHT. These criteria should be clearly defined and communicated to referring providers.

#### 3.10.2 Reminder

Even providers who view DMHT positively are likely to forget DMHT as a treatment option. Reminders can be integrated into best practice alerts, although it is also recognized that providers already suffer from alert fatigue. Other processes, such as giving providers regular feedback on their referral rates along with information about the success of the DMHTs can help providers integrate DMHT into their practices over time.

#### 3.10.3 Referral

It is recommended that the referral process be as automated as possible to reduce burden on the referrer. Electronic health records (EHRs) can be configured to allow DMHTs (with direct links) to be ordered alongside other interventions, included on a prescription list alongside medications, and/or included as a recommendation in the after-visit summary. Smartphrases can be added to the EHR, which allow providers to type short phrases that automatically insert information about the DMHT into a message to the patient through the patient portal or to the after-visit summary.

#### 3.10.4 Onboarding

Patient interest in and intention to use a DMHT frequently does not translate into patient action. Helping the patient download and register with the DMHT during their appointment will produce the largest uptake. Given provider time constraints, many pilots have introduced a digital navigator, who can assist the individual accessing the DMHT and building confidence by helping the patient feel comfortable and proficient in using the DMHT. If it is not possible to assist the patient in the office, contacting patients who do not connect to the DMHT service within 24–48 h will improve uptake (see Patient Engagement). Digital navigators can play a crucial role in digital health equity, assisting people with low digital literacy or who may have barriers to the use of the technology.

### 3.11 Patient engagement (maturity: 3-high)

#### 3.11.1 First DMHT presentation

How the provider presents the DMHT is a critical factor, particularly as many people may be unfamiliar with digital therapeutics. The workgroup agreed that the DMHT should be introduced in collaborative fashion and found the following points useful to include: (1) *Evidence:* Explaining that DMHTs are effective for the presenting condition; (2) *Description of the recommended DMHT* and what the patient's experience with the program might be; (3) *Routine care:* presenting the option as a frontline treatment option for the patient's condition in their healthcare practice; (4) *Advantages of the DMHTs for the specific patient*, such as that they are easier to fit into the patient's life as they do not require regular visits, which can be hard to schedule; (5) *Use Expectations:* Describe the expected use (e.g., the app works best if you use it X times per week); (6) *Provider's personal observations* that they have seen patients like this DMHT and benefit from it; (7) *Immediacy*, the patient can start right away; and (7) *Balance*, credibility may be enhanced by also mentioning any limitations, such as requiring some commitment to engaging with the DMHT's programs recommended activities (which is also true of psychotherapy), or any device or connectivity limitations; and (8) *Integration into care:* Letting the patient know that the provider will follow-up with the patient to see how it is going lets the patient know that the provider will stay involved and can increase the patient's motivation to use the DMHT.

While most pilots have implemented DMHTs as a first step in care, with psychotherapy as a possible next step, some have also offered DMHTs to patients in psychotherapy. Those who implemented DMHTs in a stepped manner highlighted the importance of mentioning continued care, with psychotherapy continuing to be an option. Patients who voice a preference for psychotherapy were provided a referral to psychotherapy.

#### 3.11.2 Connecting to the DMHT

Ideally, a patient would perform all registration processes before leaving the office. For example, for apps, the provider or a medical assistant would help them download and register with the DMHT. When it is not possible to register during the office visit, ta digital navigator should follow up with the patient within 24–48 h afterwards to assist, as patient motivation to initiate DMHT treatment may begin to fade over time (42). For any DMHT that requires equipment (e.g., virtual reality), shipment should be expedited, and a digital navigator should follow up with the patient upon delivery to support connecting to the program.

#### 3.11.3 Sustaining engagement

Maintaining engagement with DHMT over time has been a challenge (43). We note that this is not unique to DMHT; discontinuation rates for psychotherapy and pharmacotherapy, particularly administered through primary care, are also high (44, 45). Integrating the DMHT into processes of care improves sustained engagement with the DMHT. If providers are able to see engagement with the app and mention it at follow-up appointments or contacts, engagement will be higher. Some pilots have employed care managers or digital navigators to follow-up with patients on their use and response, both to support patient engagement and to triage non-responsive patients to appropriate levels of care (22).

#### 3.11.4 Designing referral processes

Most pilots relied on referrals from primary care or non-mental health specialty physicians, nurses, care managers, and behavioral health specialists in integrated care settings. The average time for an appointment with a primary care physician in the US 11–21 min (46), during which time there are often multiple medical problems that must be addressed, and all providers are overburdened. The referral pathways and processes to maximize patient uptake can be difficult to integrate into provider workflows. The processes described under care and referral pathways represent strategies that optimize patient uptake, but if they overtax already overburdened providers, the pilot will fail. It is essential to work with innovation-minded provider representatives (see *provider champions* below) from the clinic and if possible patient representatives in co-designing care and referral pathways to balance the use of effective referral and onboarding processes with provider capacity.

### 3.12 Training and maintaining provider and staff engagement (maturity rating: 2-moderate)

#### 3.12.1 Provider champions

There was consensus in the workgroup that identifying innovation-minded providers and champions is critical. Provider champions were involved in designing implementation plans and provided a conduit for early learnings that are part of optimization cycles, which will make the pilot successful. Their influence and enthusiasm can help shape their peers’ perspectives, and by sharing their experiences and best practices with their peers, they can help shape their peers’ practice.

#### 3.12.2 Training

Training providers and staff involved in the implementation is required to ensure that they recognize the purpose and goals of the DMHT implementation, are motivated to support the goals, and understand what is required of them for success. Training should be considered a coordinated effort addressing multiple groups because, as one workshop member put it, “You’re training a system.” Providers and staff cannot be expected to be familiar with DMHT. Digital health is not yet a formal part of medical, nursing, social work, or other clinical education programs. Most providers are not familiar with DMHT and may be skeptical.

In addition to providers, training was recommended for any staff who may interact with patients, such as medical assistants who may be asked questions or assist patients in accessing the DMHT, front desk staff who may be asked questions, and information technology, who may be involved in integrating the DMHT into the healthcare system.

Comprehensive training modules should be created in collaboration with clinician and staff representatives that include some or all of the following, depending on the person's role: (1) familiarization with this new category of mental health interventions, including the benefits relative to other treatment options; (2) objectives of the healthcare system in implementing this DMHT; (3) information on the DMHT, including how it works, how patients would use it, and the benefit for patients. Case studies can be effective in conveying this information; (4) information that instills confidence in the DMHT, such as evidence for the product both from randomized clinical trials and real-world data; (5) the intended patient population; (6) how to select patients who are good fits the DMHT; (7) the process of referring a patient to the DMHT, including scripted examples of how referrers can present the DMHT to the patient; (8) how to explain the use and benefits of the DMHT to the patient; and (9) how to interpret and utilize any data or clinical reports that may be generated by the DMHT; and (10) where the DMHT fits into the broader mental health care pathway.

A training program should be structured to occur over time as single session trainings were not seen as effective in altering long-standing workflows. Ideally, training should be conducted in person. When this is not possible for all staff groups, high quality videos, while not ideal, have been substituted. Leveraging champions from within the clinic to support and conduct ongoing training can enhance provider engagement. Subsequent training can include peer-to-peer sharing of successful practices, as well as information on how patients may have improved. Several participants mentioned it can be helpful to have company representatives present the product, as they will be more familiar with it and may have had experience implementing it in similar settings. However, because company representatives may be seen as having a conflict of interest, it was recommended that these contacts occur after an initial training and that they are facilitated by the champion or another person from within the healthcare system.

A number of strategies for encouraging training attendance were mentioned, including providing food at any meeting, providing continuing education credits for longer provider training, and building brief training into regularly occurring events such as clinic business meetings.

#### 3.12.3 Maintaining referring provider trust

The fragility of trust in the DMHT among referring providers, given DMHT is a new form of treatment, is common enough that it should be anticipated and planned for. While positive comments to the referring provider from patients referred to the DMHT can increase the provider's confidence in the DMHT, one or two negative comments from patients can quickly undermine confidence. This can be either because they are less likely to hear from satisfied patients, or due to negativity bias, a normal cognitive bias in which negative information tends to be more salient than positive information (47). This underscores the importance of providing regular feedback on the effectiveness of the DMHT to providers during the pilot.

### 3.13 Patient risk management (maturity level: 3-high)

Supporting patients on a care continuum must also consider appropriate risk management protocols. Even if a DMHT is supporting patients who are subclinical or have milder symptoms, protocols must be clear about connecting a user to crisis support (e.g., 988 or 911), or connecting back to the user's healthcare provider, should that need arise. DHMTs can provide clarity via documentation of “intended use” and “instructions for use” and via onboarding user regarding crisis escalation.

The primary risk for patients with mental health conditions is non-fatal self-harm or suicide. A number of suicide risk management guidelines exist and should be in place in healthcare settings and thus are beyond the scope of this playbook. However, in a collaboration between a healthcare system and DMHT company, it is important to ensure that when risk is detected by the company, clear roles, responsibilities, and methods of communication are agreed upon to ensure patients receive appropriate care.

#### 3.13.1 Detection

A company may receive information indicating suicide risk in a variety of ways. DMHTs commonly collect patient reported outcomes, which may include items that assess suicidality (e.g., the PHQ-9 for depression). The patient may input information into the program that indicates suicidality or may communicate suicidal risk to company staff (e.g., coaches or behavioral health specialists). While the implementations covered by the workgroup members and KOL interviewees all focused on common mental health conditions, it is nonetheless possible that other risks may arise, including mania, psychosis, or intent to harm others. These are typically not assessed in DMHTs for common mental health conditions, and therefore are most likely to be detected through clinical visits or communications with staff.

#### 3.13.2 Intervention

Most DMHTs automatically provide the patient with crisis resources (e.g., 988) when risk is detected; several participants from healthcare systems felt such automated responses are not sufficient in the context of healthcare. Companies that provide human support should be expected to have trained their staff in suicide risk management protocols to manage immediate crises.

#### 3.13.3 Clinical integration

Many participants from healthcare felt it was important to involve the healthcare system when serious risk was detected. These protocols involved the following: (1) Risk stratification, which determines which patients at which levels of risk (e.g., active suicidal ideation, ideation with a plan, ideation with a plan and intent) should be referred back to a healthcare system provider; (2) how that handoff should be made (e.g., patient is referred and/or the company contacts a designated person or service); and (3) what data or information regarding risk should be transmitted to the healthcare system and how. As this involves disclosure of PHI, these processes should be disclosed to patients prior to initiating the DMHT.

### 3.14 Human support (maturity level: 1-low)

There was consensus that companies should provide adequate technical support for the digital product for patients to be able to use or address problems that arise with digital products. Beyond that, there is considerable variability in the type of human support included in a DMHT service. While many DMHTs are fully automated, many others also use human support, which commonly use low intensity support for adherence, monitoring and feedback, or support in using skills taught by the program (48). Support can be provided by licensed clinicians, or by lay people, peers, or paraprofessionals, who typically receive training and supervision. The media used for support varies widely from messaging to video or phone calls.

Where human supporters sit has varied. While some implementations have been successful at integrating support into healthcare system workflows (22), a number of attempts at using existing staff, such as care managers in collaborative care programs, have failed because the added duties of supporting a DMHT increased their workload and did not fit easily into existing workflows. Use of human support digital navigation and adherence monitoring has been successful where staff time and workflows have been adjusted; however, this requires a significant investment in terms of people, workflow integration, and training. Alternatively, many companies that use a DMHT model that involves human support now offer both the DMHT product and the human support, allowing the healthcare system to refer out for the entire digital service. This may reduce some of the implementation burden around training and maintaining staff for the healthcare system, but may result in other complications, including managing communications between the healthcare system and company around care, and additional contractual negotiations, for example through a business associate agreement.

For all human support, the work group recommended having the following: (1) *Protocols* that provide supporters and digital navigators with clear guidance on the treatment model goals and processes, what to say and when including example scripts, and guidance on common problems and how to manage them. When possible, input from those who will be providing support services is recommended to ensure that that they are feasible and can be integrated into workflows; (2) *Sample messages* that can be copied and pasted or lightly edited for support programs that use messaging; (3) *Quality assurance* which includes training prior to beginning as well as ongoing supervision to prevent drift and ensure that the support is being provided as intended.

Workgroup members cautioned against having too many people interacting with the patient. While it may be efficient for implementers to have separate people providing digital navigation to support onboarding, technical support, and coaching or clinical support, patients usually do not understand these distinctions and can become confused.

### 3.15 Data integration (maturity: 2-moderate)

Healthcare systems usually require data from the DMHT for several purposes. Data such as usage or patient reported outcomes collected within the DMHT are often used as KPIs in the evaluation of the pilot. Providers and staff may want to see usage or outcome data from the DMHT to support its use or to integrate the DMHT into other ongoing treatments. Thus, data exchange is often part of the pilot. How data are exchanged during the pilot varies substantially.

Full integration of DMHT data into the EHR allows for seamless data sharing from the company to the healthcare system, facilitating incorporation data into patient records, patient registries, and decision support tools. However, full integration can be a long, laborious, and expensive process. Data must be standardized using interoperability standards such as Fast Healthcare Interoperability Resources (FHIR), application programming interfaces (APIs) must be built for the transfer of the data from the company to the healthcare system, and DMHT data fields must be mapped to corresponding EHR fields.

Given the cost and time involved, many pilots leave full data integration to later stages of scaling, when the healthcare system and company have more confidence that the implementation is meeting the healthcare systems goals. Instead, many pilots rely on manual transmission of requisite data using simple formats such as spreadsheets without protected health information, transferred through secure platforms or portals. However, manual data transfer can significantly limit integration into referral pathways and supports. Ensuring that relevant data reaches providers where and when they need it is difficult and time consuming, and, if not managed properly, can pose risks to the implementation. Balancing the challenges of data integration and the needs for successful implementation was a common problem, for which solutions varied considerably.

### 3.16 Privacy and security (maturity level: 2-moderate)

A collaboration between a DMHT company and a healthcare system raises issues related to data privacy and security issues. (1) *Who owns the data*, which is collected from the healthcare system's patient by the DMHT company. Given the early stage of the DMHT field, some considerations include what happens to the data in the event the company is acquired or goes bankrupt. (2) *Patient consent*. How will informed consent be obtained (and withdrawn) for data collection, use, and sharing between the company and the healthcare system? How are the terms and conditions made clear and transparent to patients? For youth, the age of consent varies across states, and a waiver of parental consent is not permitted for digital tools in some cases. (3) *Preventing unauthorized access.* How are data securely stored and transferred to protect against unauthorized access, breaches, or loss? What encryption and access controls are used? What considerations are needed for sharing data that are much higher risk (e.g., audio, video, continuous GPS)? (4) *Certification and insurance.* Healthcare systems often require several forms of assurance from companies, including HITRUST certification, which certifies security and privacy standards are met, including HIPAA, cybersecurity, and information management security. In addition, healthcare systems commonly require companies to carry cyberinsurance and coverage for indemnification.

### 3.17 Continuous quality improvement (maturity rating: 3-high)

While having a well-defined implementation plan is necessary for success, rigid implementation is unlikely to succeed. Much will go wrong, and there will be many opportunities for learning and improvement. Continuous quality improvement (CQI) is an ongoing, iterative, systematic, data-driven method of evaluating and enhancing processes and services to achieve better outcomes and performance throughout all phases of implementation. CQI processes should continue throughout the pilot and often into scaling, although intensity and frequency of CQI activities may be reduced as targets are reached. Plan-Do-Study-ACT (PDSA) is a commonly used CQI framework (49).

### 3.18 Decision to de-implement (maturity level: 2-moderate)

Decisions to de-implement or terminate the pilot can be made for several reasons, including a failure to meet the healthcare system's goals, reach KPI metrics, a change in the healthcare system's goals, or because a company is no longer able to provide the service, which can happen for a variety of reasons, including be acquired or going out of business. When the decision to terminate is made, a de-implementation plan must be developed, which can include (1) *Patient management.* If there is some flexibility, it is usually preferable to halt new referrals and allow recently referred patients to complete a course of treatment. If this is not possible, active patients should be referred to another behavioral or psychological treatment. (2) *Provider and staff management.* Providers and staff members who have been participating in the pilot should be notified and usually want to know the reasons for the termination. (3) *Technology.* All EHR tools, links, and tools related to the referral pathways, marketing, and messaging must be removed. (4) *Conduct a final review.* Conducting a final review of processes that worked or did not work can benefit both the healthcare system and the company in future DMHT implementations. This may include both quantitative data (e.g., KPIs) and qualitative data through interviews with key participants in the pilot. (5) *Media.* The termination may get picked up in the media. The healthcare system and DMHT company should develop a coordinated plan for communicating with media.

## 4 Discussion

The Society for Digital Mental Health's DMHT Implementation Workgroup engaged representatives from healthcare organizations and DMHT companies to surface their experiences with DMHT implementation, including methods used and their level of success. Based upon this information, we developed the first DMHT Implementation Playbook intended to facilitate and accelerate the successful integration of DMHTs into American healthcare organizations.

While there have only been a few reports of DMHT implementations within American healthcare systems in the scientific literature (21, 50), there is a growing literature on adapting existing implementation models to DMHTs (40, 51), which has largely used the EPIS (Exploration, Preparation, Implementation, Sustainment) framework (52) and implementation strategies from the Expert Recommendations for Implementation Change (ERIC) (53). The findings from our workgroup largely agree with and in some cases extend those recommendations those adaptations.

This playbook addresses primarily the EPIS Implementation phase. Many of the contexts and processes defined in the Exploratory and Preparation phases would have occurred prior to the processes described in this playbook, although they can shift and require adjustment during implementation. Leadership will have approved the project and funding will have been allocated, although priorities within healthcare organizations can shift. Reimbursement mechanisms remain a challenge for healthcare organizations relying on fee-for-service models, although as we have seen with the recent CMS approval of DMHT billing codes (54), this is also changing. As there was insufficient experience with Sustainment among workgroup members and KOL interviewees, this was not covered.

Our workgroup reached a high degree of consensus on the pilot and scaling stages of implementation. These stages are somewhat more nuanced than recommendations for DMHT implementation and may be more iterative than is found in many DMHT implementation trials (55–58) which, while sometimes roll out sequentially, often are testing specific implementation strategies. In contrast, our workgroup found that in real-world practice it is best to begin with a small pilot to learn how to implement, refine the strategies, and minimize risk to the healthcare system. Scaling in this context referred to scaling up to similar kinds of settings either within a regional setting or to other facilities within the same healthcare organization. We did not explore scaling out to other kinds of settings or populations (30) as the perspective was implementation with a healthcare setting. However, DMHT companies contract with a wide variety of healthcare providing organizations and could provide insights into processes for scaling out.

The importance of engaging a broad group of stakeholders to plan and maintain the implementation is widely recognized (40, 51, 53). Our workgroup found that establishing clear KPIs was a principal early task for stakeholders. While KPIs are well defined in many related fields such as health and informatics (59, 60), a recent scoping review found a significant gap in the literature on KPIs for digital health (33). This workgroup provides the first comprehensive list of KPIs for DMHT implementation. However, while we provide some examples for KPI metrics, it was beyond the scope and capacity to define the metrics. While KPI metrics depend in part on the specific and individual needs and goals of the healthcare organization, a set of well-defined, standardized KPI metrics would support implementation efforts and provide consistent data to evaluate successful implementation practices across healthcare systems.

The importance of training of clinicians and staff is consistent with the implementation literature (40, 51, 53), although the cost-effectiveness of using champions has somewhat less consistent support in the scientific literature (61). Training clinicians was seen by the workgroup and KOL interviewees as a very challenging task and input was somewhat inconsistent. The training tasks are substantial, as most clincians and staff are not familiar with digital therapeutics as a class of interventions, however clinician time is in very short supply. Different implementations have managed the challenge of training in busy clinical environments in different ways, including using in-service trainings, videos, and brief refreshers in clinic business meetings.

DMHT adaptations to implementation frameworks have stressed the importance of data integration for the management of DMHT services and measurement-based care practices (40, 51). The workgroup recommendations agree with that perspective but found that in practice full data integration is not always feasible during the pilot or early scaling phases due to cost and time. The question of when data integration should occur remains an open question. Future work identifying the determinants, such as when the cost of effort of workarounds exceeds the cost and effort of data integration will help healthcare organizations identify KPIs and criteria that can used to determine when data integration should occur.

Integration of DMHT data can have many potential benefits. Workgroup and KOL interviewees emphasized the use of data to support measurement-based care practices through regular review of data and treatment plans. DMHTs may also be able to support learning healthcare systems, in which knowledge generation processes using science, informatics, incentives, and culture are aligned to continuously improve clinical care (62). DMHTs can bring valuable real-world data into EHRs, including patient outcomes, treatment engagement patterns, and as artificial intelligence refines and validates appropriate algorithms harnessing networked sensors from phones and wearables, sensed behavioral markers related to mental health (63, 64). These data can provide near continuous information on patient response to treatments, enabling more rapid adjustment of treatment plans and reducing the time to recovery or symptom remission. This will also give healthcare systems the capacity to integrate continuous quality improvement methods for mental healthcare, leveraging data-driven insights to adapt and refine services more quickly, optimize resource allocation, and develop more personalized treatment approaches (65, 66). By leveraging these data-driven insights, healthcare organizations can adapt their services more quickly, optimize resource allocation, and develop more personalized treatment approaches, ultimately fostering a culture of continuous improvement and innovation in mental health care delivery.

A number of the areas covered in this playbook have received little to no attention to date, including criteria used to select DMHT products and companies, selection of KPIs, the recommendation to place DMHTs upstream in care pathway and not as an alternative for patients waitlisted for psychotherapy, and recommendations for how DMHTs should be introduced to patients. While these recommendations are based on the collective experiences of our workgroup members and KOL interviewees, they are nonetheless preliminary. The failures of DMHT implementations in waitlists for psychotherapy may reflect other implementation problems rather than the placement, and it is also possible that as people become more familiar with DMHTs, they may become more accepting. The processes recommended for clinician referral, and ensuring patient uptake and engagement represent the aggregation of opinions of people with experience in implementation, but have not been scientifically evaluated. These novel recommendations should therefore be interpreted with caution.

Workgroup participants and KOL participants represented a wide variety of healthcare systems and companies. Healthcare systems ranged considerably in size, as well as in structure, including value-based care systems, fee-for-service, public, and private. Companies also ranged in the types of DMHT products and in size, from smaller start-ups to multinational companies. However, healthcare systems are extremely heterogeneous, and we undoubtedly have not captured the broad range of contextual factors within each healthcare system and each DMHT that might influence how to successfully implement DMHTs. Indeed, while most healthcare systems and companies we approached were enthusiastic to participate, some did not respond to our outreach, and some declined to participate. We cannot know if the experiences of those organizations or companies that declined would have substantially changed the findings in this report. Finally, this workgroup was focused solely on the American healthcare system. Thus, while we found a fair amount of consistency at many of the methodologies used and factors seen as critical to success, we nevertheless urge that these recommendations be applied with thoughtful consideration.

Several limitations should be noted. First, these recommendations are based on the experiences reported by the workgroup members and KOL interviewees. While some of these recommendations are consistent with the implementation science literature, many go beyond the existing science. The implementations have all been for common mental health problems should not be generalized to implementations of DMHTs for severe mental illness. Most of the experiences of our workshop members and KOL participants were with pilots, scaling was less common, and few had begun to tackle long-term sustainment. This Playbook represents a snapshot in time, in which DMHT implementation in the US is just emerging and may have a limited shelf-life. We expect that as the field evolves, the methods described and factors supporting success will change. In the larger context, shifts in reimbursement mechanisms, which may be set in motion by the recent CMS DMHT billing codes, may accelerate implementation efforts. As the DMHT marketplace matures and regulatory processes become clearer, the processes and criteria for selecting DMHTs may change. Within healthcare systems, as clinicians and patients become more familiar with digital therapeutics and DMHTs, some of the implementation challenges described here may diminish. For example, as patients and providers become familiar with DMHTs, challenges engaging providers and patients may lessen. Thus, this document represents the accumulated knowledge of real-world implementers of DMHTs in healthcare at the time of this workgroup, and we expect these recommendations will change over time as scientific evidence emerges, DMHTs improve and become more widely available, and our healthcare and regulatory systems evolve.

Despite the limitations, this document represents our best knowledge at this time on how to successfully implement DMHTs in American healthcare organizations. We are on the precipice of a digital revolution in mental healthcare. With robust scientific evidence supporting the effectiveness of DMHTs (8, 9), successful implementation will take us down the final stretch to bring DMHT into the American healthcare system.

## Data Availability

The datasets presented in this article are not readily available because this was a qualitative study. Interviews were transcribed and may contain identifying information. Requests to access the datasets should be directed to the corresponding author.

## Footnotes

^1^Youn S, Schuler K, Sah P, Jaso B, Pennine M, O'Dea H, et al. Scaling out a digital-first behavioral health care model to primary care. Under Review.

## References

[1] Ettman CK Cohen GH Abdalla SM Sampson L Trinquart L Castrucci BC Persistent depressive symptoms during COVID-19: a national, population-representative, longitudinal study of U.S. adults. Lancet Reg Health Am. (2022) 5:100091. 10.1016/j.lana.2021.100091 34635882 PMC8488314

[2] Vahratian A Blumberg SJ Terlizzi EP Schiller JS. Symptoms of anxiety or depressive disorder and use of mental health care among adults during the COVID-19 pandemic - United States, August 2020-February 2021. MMWR Morb Mortal Wkly Rep. (2021) 70(13):490–4. 10.15585/mmwr.mm7013e2 33793459 PMC8022876

[3] Substance Abuse and Mental Health Services Administration. Key Substance Use and Mental Health Indicators in the United States: Results from the 2021 National Survey on Drug Use and Health (HHS Publication No. PEP22-07-01-005, NSDUH Series H-57) Washington, DC: Substance Abuse and Mental Health Services Administration. (2022). Available online at: https://www.samhsa.gov/data/report/2021-nsduh-annual-national-report

[4] Pierce BS Perrin PB Tyler CM McKee GB Watson JD. The COVID-19 telepsychology revolution: a national study of pandemic-based changes in U.S. Mental health care delivery. Am Psychol. (2021) 76(1):14–25. 10.1037/amp0000722 32816503

[5] Cantor J McBain RK Kofner A Hanson R Stein BD Yu H. Telehealth adoption by mental health and substance use disorder treatment facilities in the COVID-19 pandemic. Psychiatr Serv. (2022) 73(4):411–7. 10.1176/appi.ps.202100191 34407631 PMC10695271

[6] Health Services and Resources Administration. Health Workforce Shortage Areas. Washington, DC: U.S. Department of Health and Human Services (2024). Available online at: https://data.hrsa.gov/topics/health-workforce/shortage-areas

[7] Torous J Bucci S Bell IH Kessing LV Faurholt-Jepsen M Whelan P The growing field of digital psychiatry: current evidence and the future of apps, social media, chatbots, and virtual reality. World Psychiatry. (2021) 20(3):318–35. 10.1002/wps.20883 34505369 PMC8429349

[8] Linardon J Torous J Firth J Cuijpers P Messer M Fuller-Tyszkiewicz M. Current evidence on the efficacy of mental health smartphone apps for symptoms of depression and anxiety. A meta-analysis of 176 randomized controlled trials. World Psychiatry. (2024) 23(1):139–49. 10.1002/wps.21183 38214614 PMC10785982

[9] Moshe I Terhorst Y Philippi P Domhardt M Cuijpers P Cristea I Digital interventions for the treatment of depression: a meta-analytic review. Psychol Bull. (2021) 147(8):749–86. 10.1037/bul0000334 34898233

[10] Leung C Pei J Hudec K Shams F Munthali R Vigo D. The effects of nonclinician guidance on effectiveness and process outcomes in digital mental health interventions: systematic review and meta-analysis. J Med Internet Res. (2022) 24(6):e36004. 10.2196/36004 35511463 PMC9244656

[11] Clarke S Hanna C Mulholland C Shannon C Urquhart C. A systematic review and meta-analysis of digital health technologies effects on psychotic symptoms in adults with psychosis. Psychosis. (2019) 11(4):362–73. 10.1080/17522439.2019.1632376

[12] Anmella G Faurholt-Jepsen M Hidalgo-Mazzei D Radua J Passos IC Kapczinski F Smartphone-based interventions in bipolar disorder: systematic review and meta-analyses of efficacy. A position paper from the international society for bipolar disorders (ISBD) big data task force. Bipolar Disord. (2022) 24(6):580–614. 10.1111/bdi.13243 35839276 PMC9804696

[13] Kim SK Lee M Jeong H Jang YM. Effectiveness of mobile applications for patients with severe mental illness: a meta-analysis of randomized controlled trials. Jpn J Nurs Sci. (2022) 19(3):e12476. 10.1111/jjns.12476 35174976

[14] Schueller SM Hunter JF Figueroa C Aguilera A. Use of digital mental health for marginalized and underserved populations. Curr Treat Options Psychiatry. (2019) 6:243–55. 10.1007/s40501-019-00181-z

[15] Harty S Enrique A Akkol-Solakoglu S Adegoke A Farrell H Connon G Implementing digital mental health interventions at scale: one-year evaluation of a national digital CBT service in Ireland. Int J Ment Health Syst. (2023) 17(1):29. 10.1186/s13033-023-00592-9 37817270 PMC10563351

[16] Goeldner M Gehder S. Digital health applications (DiGAs) on a fast track: insights from a data-driven analysis of prescribable digital therapeutics in Germany from 2020 to mid-2024. J Med Internet Res. (2024) 26:e59013. 10.2196/59013 39208415 PMC11393499

[17] Hopkin G Branson R Campbell P Coole H Cooper S Edelmann F Building robust, proportionate, and timely approaches to regulation and evaluation of digital mental health technologies. Lancet Digit Health. (2024) 7(1):E89–93. 10.1016/S2589-7500(24)00215-2 39550311 PMC7618730

[18] Nomura A. Digital therapeutics in Japan: present and future directions. J Cardiol. (2024) in press. 10.1016/j.jjcc.2024.11.005 39613155

[19] Titov N Dear BF Nielssen O Wootton B Kayrouz R Karin E User characteristics and outcomes from a national digital mental health service: an observational study of registrants of the Australian MindSpot clinic. Lancet Digit Health. (2020) 2(11):e582–e93. 10.1016/S2589-7500(20)30224-7 33103097 PMC7571905

[20] Mohr DC Azocar F Bertagnolli A Choudhury T Chrisp P Frank R Banbury forum consensus statement on the path forward for digital mental health treatment. Psychiatr Serv. (2021) 72(6). 10.1176/appi.ps.202000561 33467872 PMC8822332

[21] Mordecai D Histon T Neuwirth E Heisler S Kraft A Bang Y How Kaiser permanente created a mental health and wellness digital ecosystem. NEJM Catalyst. (2021) 2(1). 10.1056/CAT.20.029534514431

[22] Youn SJ Jaso B Eyllon M Sah P Hoyler G Barnes JB Leveraging implementation science to integrate digital mental health interventions as part of routine care in a practice research network. Adm Policy Ment Health. (2024) 51(3):348–57. 10.1007/s10488-023-01292-9 37615809

[23] Hsieh HF Shannon SE. Three approaches to qualitative content analysis. Qual Health Res. (2005) 15(9):1277–88. 10.1177/1049732305276687 16204405

[24] Proctor EK Powell BJ McMillen JC. Implementation strategies: recommendations for specifying and reporting. Implement Sci. (2013) 8:139. 10.1186/1748-5908-8-139 24289295 PMC3882890

[25] Glasgow RE Harden SM Gaglio B Rabin B Smith ML Porter GC RE-AIM planning and evaluation framework: adapting to new science and practice with a 20-year review. Front Public Health. (2019) 7:64. 10.3389/fpubh.2019.00064 30984733 PMC6450067

[26] Institute for Healthcare Improvement. Quality Improvement Essentials Toolkit: Insitute for Healthcare Improvement. Boston, MA: Institute for Healthcare Improvement (2024). Available online at: https://www.ihi.org/resources/tools/quality-improvement-essentials-toolkit

[27] Digital Therapeutics Alliance. DTX Evaluation Toolkit. Digital Therapeutics Alliance (2023). Available online at: https://dtxalliance.org/understanding-dtx/dtx-evaluation-toolkit/

[28] Kachirskaia I Mate KS Neuwirth E. Human-centered cesign and performance improvement: better together. NEJM Catalyst. (2018). 10.1056/CAT.18.0144

[29] Schlieter H Marsch LA Whitehouse D Otto L Londral AR Teepe GW Scale-up of digital innovations in health care: expert commentary on enablers and barriers. J Med Internet Res. (2022) 24(3):e24582. 10.2196/24582 35275065 PMC8956989

[30] Aarons GA Sklar M Mustanski B Benbow N Brown CH. “Scaling-out” evidence-based interventions to new populations or new health care delivery systems. Implement Sci. (2017) 12(1):111. 10.1186/s13012-017-0640-6 28877746 PMC5588712

[31] Chambers DA Glasgow RE Stange KC. The dynamic sustainability framework: addressing the paradox of sustainment amid ongoing change. Implement Sci. (2013) 8:117. 10.1186/1748-5908-8-117 24088228 PMC3852739

[32] Gagliardi AR Marshall C Huckson S James R Moore V. Developing a checklist for guideline implementation planning: review and synthesis of guideline development and implementation advice. Implement Sci. (2015) 10:19. 10.1186/s13012-015-0205-5 25884601 PMC4329197

[33] Brenner M Weir A McCann M Doyle C Hughes M Moen A Development of the key performance indicators for digital health interventions: a scoping review. Digit Health. (2023) 9:20552076231152160. 10.1177/20552076231152160 36714542 PMC9880566

[34] Weiner BJ Lewis CC Stanick C Powell BJ Dorsey CN Clary AS Psychometric assessment of three newly developed implementation outcome measures. Implement Sci. (2017) 12(1):108. 10.1186/s13012-017-0635-3 28851459 PMC5576104

[35] Glasgow RE Vogt TM Boles SM. Evaluating the public health impact of health promotion interventions: the RE-AIM framework. Am J Public Health. (1999) 89(9):1322–7. 10.2105/AJPH.89.9.1322 10474547 PMC1508772

[36] Proctor E Silmere H Raghavan R Hovmand P Aarons G Bunger A Outcomes for implementation research: conceptual distinctions, measurement challenges, and research agenda. Adm Policy Ment Health. (2011) 38(2):65–76. 10.1007/s10488-010-0319-7 20957426 PMC3068522

[37] Schrijvers G van Hoorn A Huiskes N. The care pathway: concepts and theories: an introduction. Int J Integr Care. (2012) 12:e192. 10.5334/ijic.812 23593066 PMC3602959

[38] Barbar J Bistline K Dubenitz J Everett A Humensky J Katz I Use of measurement based care for behavioral health. Report from Substance Use and Mental Health Services Administration, Washington, DC. (2022). Available online at: https://www.samhsa.gov/sites/default/files/ismicc-measurement-based-care-report.pdf

[39] Lewis CC Boyd M Puspitasari A Navarro E Howard J Kassab H Implementing measurement-based care in behavioral health: a review. JAMA Psychiatry. (2019) 76(3):324–35. 10.1001/jamapsychiatry.2018.3329 30566197 PMC6584602

[40] Graham AK Lattie EG Powell BJ Lyon AR Smith JD Schueller SM Implementation strategies for digital mental health interventions in health care settings. Am Psychol. (2020) 75(8):1080–92. 10.1037/amp0000686 33252946 PMC7709140

[41] Collins C Hewson DL Munger R Wade T. Evolving Models of Behavioral Health Integration in Primary Care. New York: Milbank Memorial Fund (2010).

[42] Jaso-Yim B Eyllon M Sah P Pennine M Welch G Schuler K Evaluation of the impact of a digital care navigator on increasing patient registration with digital mental health interventions in routine care. Internet Interv. (2024) 38:100777. 10.1016/j.invent.2024.100777 39410952 PMC11474210

[43] Lipschitz JM Pike CK Hogan TP Murphy SA Burdick KE. The engagement problem: a review of engagement with digital mental health interventions and recommendations for a path forward. Curr Treat Options Psych. (2023) 10:119–35. 10.1007/s40501-023-00297-3 PMC10883589 38390026

[44] Swift JK Greenberg RP Tompkins KA Parkin SR. Treatment refusal and premature termination in psychotherapy, pharmacotherapy, and their combination: a meta-analysis of head-to-head comparisons. Psychotherapy (Chic). (2017) 54(1):47–57. 10.1037/pst0000104 28263651

[45] Sansone RA Sansone LA. Antidepressant adherence: are patients taking their medications? Innov Clin Neurosci. (2012) 9(5-6):41–6. https://pmc.ncbi.nlm.nih.gov/articles/PMC3398686/pdf/icns_9_5-6_41.pdf 22808448 PMC3398686

[46] Irving G Neves AL Dambha-Miller H Oishi A Tagashira H Verho A International variations in primary care physician consultation time: a systematic review of 67 countries. BMJ Open. (2017) 7(10):e017902. 10.1136/bmjopen-2017-017902 29118053 PMC5695512

[47] Rozin P Royzman EB. Negativity bias, negativity dominance, and contagion. Pers Soc Psychol Rev. (2001) 5:296–320. 10.1207/S15327957PSPR0504_2

[48] Lattie EG Stiles-Shields C Graham AK. An overview of and recommendations for more accessible digital mental health services. Nat Rev Psychol. (2022) 1(2):87–100. 10.1038/s44159-021-00003-1 38515434 PMC10956902

[49] Leis JA Shojania KG. A primer on PDSA: executing plan-do-study-act cycles in practice, not just in name. BMJ Qual Saf. (2017) 26(7):572–7. 10.1136/bmjqs-2016-006245 27986900

[50] Nordberg SS Jaso-Yim BA Sah P Schuler K Eyllon M Pennine M Evaluating the implementation and clinical effectiveness of an innovative digital first care model for behavioral health using the RE-AIM framework: quantitative evaluation. J Med Internet Res. (2024) 26:e54528. 10.2196/54528 39476366 PMC11561446

[51] Liu M Schueller SM. Moving evidence-based mental health interventions into practice: implementation of digital mental health interventions. Curr Opin Psychiatry. (2023) 10:333–45. 10.1007/s40501-023-00298-2

[52] Aarons GA Hurlburt M Horwitz SM. Advancing a conceptual model of evidence-based practice implementation in public service sectors. Adm Policy Ment Health. (2011) 38(1):4–23. 10.1007/s10488-010-0327-7 21197565 PMC3025110

[53] Powell BJ Waltz TJ Chinman MJ Damschroder LJ Smith JL Matthieu MM A refined compilation of implementation strategies: results from the expert recommendations for implementing change (ERIC) project. Implement Sci. (2015) 10:21. 10.1186/s13012-015-0209-1 25889199 PMC4328074

[54] Centers for Medicare and Medicaid Services. Medicare Physician Fee Schedule Final Rule Summary: CY 2025. Washington, DC: Center for Medicare and Medicaid Services (2024).

[55] Gilbody S Brabyn S Lovell K Kessler D Devlin T Smith L Telephone-supported computerised cognitive-behavioural therapy: rEEACT-2 large-scale pragmatic randomised controlled trial. Br J Psychiatry. (2017) 210(5):362–7. 10.1192/bjp.bp.116.192435 28254959

[56] Gilbody S Littlewood E Hewitt C Brierley G Tharmanathan P Araya R Computerised cognitive behaviour therapy (cCBT) as treatment for depression in primary care (REEACT trial): large scale pragmatic randomised controlled trial. Br Med J. (2015) 351:h5627. 10.1136/bmj.h5627 26559241 PMC4641883

[57] Buhrmann L Schuurmans J Ruwaard J Fleuren M Etzelmuller A Piera-Jimenez J Tailored implementation of internet-based cognitive behavioural therapy in the multinational context of the ImpleMentAll project: a study protocol for a stepped wedge cluster randomized trial. Trials. (2020) 21(1):893. 10.1186/s13063-020-04686-4 33115545 PMC7592568

[58] Kleiboer A Smit J Bosmans J Ruwaard J Andersson G Topooco N European COMPARative effectiveness research on blended depression treatment versus treatment-as-usual (E-COMPARED): study protocol for a randomized controlled, non-inferiority trial in eight European countries. Trials. (2016) 17(1):387. 10.1186/s13063-016-1511-1 27488181 PMC4972947

[59] Farinango CD Benavides JS Ceron JD Lopez DM Alvarez RE. Human-centered design of a personal health record system for metabolic syndrome management based on the ISO 9241-210:2010 standard. J Multidiscip Healthc. (2018) 11:21–37. 10.2147/JMDH.S150976 29386903 PMC5767088

[60] Gartner JB Lemaire C. Dimensions of performance and related key performance indicators addressed in healthcare organisations: a literature review. Int J Health Plann Manage. (2022) 37(4):1941–52. 10.1002/hpm.3452 35288968

[61] Santos WJ Graham ID Lalonde M Demery Varin M Squires JE. The effectiveness of champions in implementing innovations in health care: a systematic review. Implement Sci Commun. (2022) 3(1):80. 10.1186/s43058-022-00315-0 35869516 PMC9308185

[62] Easterling D Perry AC Woodside R Patel T Gesell SB. Clarifying the concept of a learning health system for healthcare delivery organizations: implications from a qualitative analysis of the scientific literature. Learn Health Syst. (2022) 6(2):e10287. 10.1002/lrh2.10287 35434353 PMC9006535

[63] Mohr DC Zhang M Schueller SM. Personal sensing: understanding mental health using ubiquitous sensors and machine learning. Annu Rev Clin Psychol. (2017) 13:23–47. 10.1146/annurev-clinpsy-032816-044949 28375728 PMC6902121

[64] Meyerhoff J Liu T Kording KP Ungar LH Kaiser SM Karr CJ Evaluation of changes in depression, anxiety, and social anxiety using smartphone sensor features: longitudinal cohort study. J Med Internet Res. (2021) 23(9):e22844. 10.2196/22844 34477562 PMC8449302

[65] Bond RR Mulvenna MD Potts C O'Neill S Ennis E Torous J. Digital transformation of mental health services. NPJ Ment Health Res. (2023) 2(1):13. 10.1038/s44184-023-00033-y 38609479 PMC10955947

[66] Lim CT Fuchs C Torous J. Integrated digital mental health care: a vision for addressing population mental health needs. Int J Gen Med. (2024) 17:359–65. 10.2147/IJGM.S449474 38318335 PMC10840519

