# Supplementary material for: Digital mental health treatment implementation playbook: successful practices from implementation experiences in American healthcare organizations
Source: Front Digit Health. 2025 Feb 11;7:1509387. doi: 10.3389/fdgth.2025.1509387 (PMC11850340; doi:10.3389/fdgth.2025.1509387)
Supplement: Supplementary file 1 [file Supplementaryfile1.pdf]

Industry Implementation Interview Guide

Are you OK if I record this interview?
All responses will be aggregated. No identifying information will be included in any resulting documents.

Company History
What year was your company founded?

What funding stage is your company in? (Series stage - Seed, A, B, C, D, IPO)

What roles do clinicians play in your company?
- Do you have clinicians in C- suite roles?

How are clinician roles and representation used in developing relationships with healthcare clients?

Patient Population
What patient/user populations do you serve?

What modalities does your company offer as far as digital mental health tools (self guided/coaches/teletherapy/AI chatbot/stepped care/blended approach/other)?

Product portfolio?
Are any of the products in your portfolio FDA certified?
- Or are you in the certification process?
- Why?

Have your solutions been certified/endorsed by other organizations(FDA/ APA/ORCHA/Psyberguide/others)?

Design
How do you incorporate the patient and clinician voice in your portfolio?
How do you design for health equity?
How do you address informed consent?
What is your privacy policy?
-

If a user needs technical support, what is available to them?
How do you communicate the intended use of the product to the client and the user?

Clients (for international companies, only US focused healthcare)
What types of healthcare clients do you have experience working with?
-Payers, Providers, Health systems, hospitals etc.

What are some of the challenges when partnering with different health care clients?

Can you reflect on the sales cycles for different healthcare clients (sales reimbursement remains a challenge for DMH)
- How long has contracting taken?

- What are some of the hurdles, challenges, and barriers you've encountered?

Can you speak to your phases of deployment (pilot/spread/scale/sustain)? Does that differ by the type of healthcare client you work with?

How do you determine healthcare client readiness for implementation?

Implementation
What stages of implementation has your company had experience with (pilot/spread/scale/sustain)?

- How has your company been involved in the implementation?
  - Who did you interact with and how in the healthcare system (leadership, clinical managers, providers, IT, etc.)
- What challenges have you experienced at each stage?
  - Pilot (probe on not getting past the pilot stage)
  - Spread and scale
  - Sustain
- What are the opportunities in each of those phases? What does it take to succeed in each phase?

How do you assess how your tools will fit into workflow (workforce, hardware, software) from pilot to scale up?

- Does it fit into existing infrastructure or did it require changes to be made, and if so what?
- Did it require investment from the healthcare client?

Have you seen changes in healthcare clients' overall understanding and perceptions of DMH post pandemic?
Have expectations for what your product can do changed? If so, how?

Training
How do you approach building your training materials to support deployments? Provider facing? Patient facing?
Do you have a content review process in place to ensure clinical appropriateness and fidelity?

- When does that take place? (initial design vs. ongoing processes in place)

Reporting/Data/Protection
What data integration capabilities do you have?

- Do you allow direct data exchange (e.g. EMR ingest PROs or engagement data)- how do you account for data exchange with other systems on a technical level and policy level, with clinicians and users/patient
- How do you support data integration and interoperability?

What do your healthcare clients seek re integration?

- What reporting capabilities do healthcare clients require?

What is your most common integration starting point?
How is the the user informed that their data will be transferred to the healthcare system?

How is your solution compliant with relevant data protection laws (HIPAA) and relevant laws

- What challenges have you had in ensuring and demonstrating security?

Evidence
Can you outline your clinical evidence generation process, how has it evolved over time? (RCTs, real world evidence, health economics outcome research).
What are healthcare clients looking for regarding evidence?

Technical Risk
How do you address risk for user safety? (data servers location, use of AWS or like service, how data is stored PII/PHI, who had access to that data? What policies do you have to support these aspects of your business.
Have you obtained certifications? E.g. Soc 2, Hi-Trust etc

Ethics
How do you ensure ethical practices along and across the product cycles?

- Is there anyone else in your company you'd suggest we talk with. (Particularly for areas that the interviewee has less expertise or knowledge)
