# Supplementary material for: Digital mental health treatment implementation playbook: successful practices from implementation experiences in American healthcare organizations
Source: Front Digit Health. 2025 Feb 11;7:1509387. doi: 10.3389/fdgth.2025.1509387 (PMC11850340; doi:10.3389/fdgth.2025.1509387)
Supplement: Supplementary file 2 [file Supplementaryfile2.pdf]

Healthcare Implementation Interview Guide

Are you OK if I record this interview?
All responses will be aggregated. No identifying information will be included in any resulting documents.

- Can you describe your current role in [company/organization]? How long have you been in that role?
- What was the pain point or problem that got your healthcare system to consider digital mental health?
    - What were you trying to achieve?

Planning

- How do you choose DMH products/services/companies? (e.g., scientific evidence, reliability/performance/patient population)
    - What criteria and/or processes do you use?
- How do you typically measure success/impact when implementing a new program/product?
    - Do you use any specific evaluation frameworks do you use (if any)? (RE-AIM, Human Centered Design, Performance Improvement, others)
    - How do you use them?

Implementation

- Implementation strategies & stage (pilot, spread, scale, sustain)
    - Are you able to tell us which product you are implementing?
    - What processes were used for implementation planning?
        - Who was engaged and how (e.g. leadership, providers, IT, Quality, etc. etc.)?
            - How did you manage challenges as they came up in the implementation process? Who was involved?
        - What, in broad strokes, was the initial implementation plan?
        - What stage of implementation are you in? (pilot/spread/scale/sustain)
            - probe on how long those stages lasted
    - How was the DMH service introduced into the care pathway? (E.g. primary care, specialty behavioral health etc)
        - What challenges were encountered, and how did you manage or solve them?
    - What metrics are tracked for implementation?
        - Did you have benchmarks/quality metrics for successful implementation? If so, what were they?

Patient and Provider Engagement

- How are patients connected to service (referral pathways)?
    - What challenges have you experienced?
        - Who is needed on the healthcare side (e.g. provider referral, care manager, digital navigator). How were they engaged? What did they need to do?
    - What adaptations or changes to the referral process were made over time?
    - What solutions worked and did not work?
    - How well are your current solutions working?
    - Are there plans to continue improving and if so, what are they?

  - How is patient engagement maintained?
    - Challenges experienced
    - What adaptations or changes were made over time as far as engagement strategies
    - What solutions worked and did not work.
    - How well are your current solutions working?
      - Are there plans to continue improving and if so, what are they?

Patient Management

  - Did you use human support (coaching, care management, etc.)?
    - Why did you decide to use/not use human support?
    - Who provided the support, how, and with what frequency?
    - What if any supervision or quality assurance was used?
    - What challenges occurred, how was the process modified over time?
  - What kinds of risk management protocols were in place (e.g. suicidality, deterioration, etc.)
  - Was there a process for managing patients who did not experience symptom improvement (e.g. triaging patients)?
    - What criteria were used (to measure those who are not responding)?
    - What was the process (e.g. referral, case management, follow-up, etc.)?
  - Is there anything else about the implementation that we did not cover that you feel is important - either challenges or successes?

Data

- Data exchange/management
  - What data are exchanged between healthcare clients and the DMH company, and how? (API, CSV file etc.)
  - How are these data used (e.g. patient management, quality metrics, reimbursement, payment for digital mental health product, establishing ROI)?
  - How are data security and privacy managed?
    - What risks had to be managed?
      - Transfer of data between company and healthcare (PII transferred to company, e.g. Name, phone number)
      - Integrating high risk populations into healthcare
      - Integration of mental health data into EHRs
      - Cybersecurity issues (e.g., insurance)
    - Do patients have the option to have data removed?
  - Was this already in place, or did it need to be set up by the health system? (was the data exchange and reporting infrastructure already in place, or was it built for deployment)

Equity

- What considerations were there around equitable provision of digital mental health services?
  - Have services been tailored in any way to meet the needs of specific subpopulations or cultural groups? If so, what?
  - If services have been tailored, how were decisions made around tailoring?

Business Decisions

- Can you speak to the business value of digital mental health tools?
  - What payment models are used [per user, per license (e.g. X number of users), value-based, ROI (how is it defined), HEOR (health economic outcome research) data reporting
  - Are you tracking relevant mental health quality metrics?

            - Is this DMH deployment intended to affect those quality metrics?
    - Is it considered a standard of care?
- Has there been a decision to end a DMH implementation?
    - What factors were involved in that decision? (funding, failure to meet goals, etc.)

- Is there anyone else in your organization you'd suggest we talk with to understand implementation of digital mental health tools? (Particularly for areas that the interviewee has less expertise or knowledge)
